# Community memory screening as a strategy for recruiting older adults into Alzheimer’s disease research

**DOI:** 10.1186/s13195-020-00643-0

**Published:** 2020-06-30

**Authors:** Guerry M. Peavy, Cecily W. Jenkins, Emily A. Little, Christina Gigliotti, Amanda Calcetas, Steven D. Edland, James B. Brewer, Douglas Galasko, David P. Salmon

**Affiliations:** 1 Department of Neurosciences, University of California, San Diego, 9500 Gilman Drive, La Jolla, CA 92093-0948 USA; 2 Department of Neurology, Alzheimer Therapeutic Research Institute, University of Southern California, 9860 Mesa Rim Road, San Diego, CA 92121 USA; 3 Department of Family Medicine & Public Health, University of California, San Diego, 9500 Gilman Drive, La Jolla, CA 92093 USA; 4 Department of Radiology, University of California, San Diego, 9500 Gilman Drive, La Jolla, CA 92093 USA

**Keywords:** Dementia, Alzheimer’s disease, Memory, Cognition, Screening, Recruitment

## Abstract

**Background:**

Growing awareness of Alzheimer’s disease (AD) has prompted a demand for quick and effective ways to screen for memory loss and cognitive decline in large numbers of individuals in the community. Periodic Memory Screening Day events provide free, brief cognitive screening aimed at those 65 years and older, and can serve as an opportunity to gauge participants’ attitudes towards AD research and recruit them into ongoing research projects.

**Methods:**

Over 6 single-day events in 2 years, more than 574 individuals were individually screened using the MoCA and a story recall task (immediate and delayed), given feedback about their performance, and introduced to AD research and opportunities to participate.

**Results:**

Screening classified 297 individuals (52.0%) as having “No Decline,” 192 (33.6%) as “Possible decline,” and 82 (14.4%) as “Likely decline.” Those with “Likely decline” were older and less educated, had more memory concerns, were more likely to be men, and were less likely to have a positive family history of dementia than those with “No Decline.” Subsequent validation of screening procedures against a full clinical evaluation showed 72% classification accuracy with a skew towards over-calling *Possible* and *Likely* decline and thereby guiding questionable individuals to a more thorough evaluation. Of those screened, 378 (66%) agreed to additional research and consented to being listed in a research registry, and a majority (70–85%) of those consenting reported they were amenable to various AD research procedures including lumbar puncture, MRI, and autopsy. Overall, 19.1% of those screened met inclusion criteria for ongoing studies and were successfully recruited into AD research.

**Conclusions:**

Conducting a few concentrated community memory screening events each year may help meet the public’s demand for brief assessment of memory concerns and can be a relatively effective and efficient recruitment strategy for AD research.

## Background

Alzheimer’s disease (AD) is an age-associated neurodegenerative disease that causes insidious onset of memory loss and cognitive decline that slowly progresses to severe dementia with loss of independence in daily function. Current estimates are that 4.7 million people in the USA suffer from AD and the number is projected to increase to 13.8 million by 2050 [1]. Many millions more are impacted by the disease through a positive family history or the presence of a genetic risk factor (e.g., the ε4 allele of Apolipoprotein E (APOE)) [2, 3]. This pervasiveness has led to a growing awareness of the disease and increased concern about memory loss and its long-term consequences, particularly in the elderly and those with other AD risk factors.

Increasing concern over AD has prompted a demand for quick and effective ways to screen for memory loss and cognitive decline in large numbers of individuals in the community. To address this demand, the Alzheimer’s Foundation of America initiated an annual National Memory Screening Day to encourage clinicians/researchers nationwide to offer free memory screening to the public on a specified day each year [4]. These events are generally aimed at those over the age of 65 with concern about their memory and involve brief cognitive testing with feedback that either reassures the participant of normal function or informs them of a potential problem and the need to consult their usual healthcare provider for further evaluation. While there is some controversy about the need for widespread and somewhat indiscriminate screening for memory impairment [5, 6], identifying potential problems early in their course may have the advantage of allowing “next step” opportunities to occur, such as referral to specialized healthcare providers, identification of treatable causes of cognitive decline (i.e., [7]), and education about beneficial changes in lifestyle [8, 9].

Community screening also provides an opportunity to educate participants about AD and the importance of research. Recruitment of research volunteers is one of the most difficult and important aspects of conducting studies that explore underlying causes of AD dementia or that evaluate symptomatic treatments or disease modifying interventions [10, 11]. Without sufficient numbers of appropriate volunteers, studies may be forced to rely on samples that are not representative of the affected population or too small to support statistically meaningful conclusions [12, 13]. Therefore, it is crucial to develop strategies for timely outreach, engagement, and recruitment of research volunteers [10]. Community memory screening may be an effective strategy since it provides direct engagement between clinicians/researchers and those with memory concerns. Furthermore, it may be particularly useful for recruiting cognitively normal older adults with risk factors for AD (e.g., family history or APOE ε4 genotype) since family history is a frequent reason for concern that motivates individuals to seek memory screening [14]. It may also be useful for recruiting those with mild cognitive decline but no significant decline in function (i.e., mild cognitive impairment [15];) in instances where cognitive problems have not yet risen to a level where medical help has been sought.

As part of outreach and recruitment efforts, the UCSD Shiley-Marcos Alzheimer’s Disease Research Center (ADRC) conducted periodic Memory Screening Day events over the past 2 years that provided free brief cognitive screening aimed at those 65 years and older. The events were used as an opportunity to inform participants about cognitive decline and AD, learn about their concerns and attitudes towards AD research, and potentially recruit them into ongoing research projects. This allowed us to address questions such as the following: (1) Are those who come to be screened objectively impaired or simply “worried well”? (2) Are they appropriate for participation in AD research, including clinical trials? (3) Are they amenable to research and specific research procedures that have some degree of invasiveness or discomfort (e.g., clinical trials, lumbar puncture, autopsy)?

Here, we report the demographic characteristics of individuals who participated in Memory Screening Day events and present information on memory concerns expressed by participants, their family history of dementia, and their amenability to AD research participation. We show the proportion of screened participants who were deemed to have likely memory/cognitive decline, possible memory/cognitive decline, or no memory/cognitive decline based on objective tests of memory and global cognitive status, and provide data on the validity of our screening method. Characteristics of those who agreed or did not agree to further contact about potential research participation were compared, and likelihood of participation in specific research procedures (e.g., MRI, autopsy) was gauged in those who agreed. Finally, the value of the events as an opportunity to recruit elderly individuals with or without concerns about their memory into the ongoing ADRC longitudinal observational study or affiliated AD studies and clinical trials was examined.

## Methods

The date and location of a free “memory screening” event at the UCSD Shiley-Marcos ADRC was announced in print and on-line versions of an English language newspaper article on aging and brain health published by the San Diego Union-Tribune in early January 2017 and again in early January 2018. The announcements noted that screening was available for any adult 65 years of age or older with concerns about his or her memory. A phone number was provided for contacting the ADRC to schedule a screening appointment. Due to the enthusiastic response (e.g., over 150 calls on the day after the first article), three separate “Memory Screening Day” events were scheduled each year to accommodate the demand. These events occurred in January, April, and July 2017, and January, May, and September 2018. Each event occurred during a single day that could accommodate up to 120 participants. Screening appointments were made on a first-come, first-served basis with the appointments rolling to the next event once the first event of the year was fully scheduled (although we scheduled 132 individuals for one of the events with anticipation of “no shows”). The overflow events continued to be advertised through the ADRC newsletter and ADRC website. Three events were able to accommodate all of those who expressed a desire to be screened in a given year. Screening required 30 min and was carried out individually. Eight screens were simultaneously performed by eight raters in separate testing rooms, with each rater completing 13 to 15 screens during the event. This accommodated a total of 104 to 120 participants during each event.

### Initial contact

When participants called to inquire or make an appointment, an ADRC staff member answered general questions, obtained their age and contact information, and scheduled a specific time for screening. A map with detailed directions to the screening location at the UCSD ADRC and instructions for free parking were sent to the participant. The staff member called the participant close to their appointment date (2–3 days before) to remind them of the time and place.

### Screening visit

Prior to cognitive testing, informed consent was obtained from each participant for use of screening data for research purposes. Consent was obtained by the rater who performed cognitive testing. During the consent process, participants were made aware that their data would be de-identified and kept confidential and that they could withdraw consent for research at any time. They were also informed that the results and information provided through screening were preliminary and educational in nature and intended to provide information to facilitate a meaningful discussion with their physician or other qualified healthcare professional. They were told that the results were not intended to provide a diagnosis or recommendation for treatment or rehabilitation and do not and should not take the place of talking with their physician or other qualified healthcare professional. They were also told that if they declined to consent to have their memory screening data used for research purposes, they would still be screened and given the results, but the data would not be saved or used in any analyses. The research protocol was reviewed and approved by the UCSD Human Research Protection Program (HRPP)/Institutional Review Board (IRB).

Participants then completed screening cognitive testing and a demographics questionnaire (i.e., date of birth, years of education, gender), a language history questionnaire, and a brief, 10-point self-report scale of memory concerns (i.e., Memory Concerns Questionnaire; MCQ). Finally, participants were offered the opportunity to receive additional debriefing about their screening results, information about resources for those with memory concerns, and information about opportunities for participation in research related to memory disorders and aging. They were also asked to complete additional questionnaires about their potential willingness to participate in various types of research and research procedures.

### Rater selection and training

Raters included ADRC psychometrists and designated research assistants from local scientific collaborators who draw on ADRC research participants for their studies. All raters completed a 4-h training session with a senior psychometrist that involved a discussion of general testing protocol, strategies to reassure and encourage participants who are anxious, a review of Health Insurance Portability and Assurance Act (HIPAA) requirements, and detailed administration and scoring training on the neuropsychological tests used in the screening process. There was also detailed training on the use of the algorithm needed to complete the feedback letter (see below). Bilingual staff members with demonstrated Spanish-English fluency were available to provide testing in either language, depending on the participant’s self-reported preference.

### Cognitive testing

Each participant was tested in a quiet, well-lit room at the Shiley-Marcos ADRC by a trained rater. The screening battery consisted of the Montreal Cognitive Assessment (MoCA) scale [16] and the immediate and delayed recall conditions of Story A from the Logical Memory subtest of the Wechsler Memory Scale-Revised (WMS-R) [17]. The MoCA is a brief mental status exam that assesses attention, concentration, executive functions, memory, language, visual constructional skills, conceptual thinking, calculations, and orientation. Scores range from 0 to 30, with higher scores indicating better performance. An education adjustment is built into the MoCA so that 1 point is added to the total score for those with 12 years of education or less. The WMS-R Logical Memory, Story A is a measure of verbal episodic memory. A short, one-paragraph story that consists of 25 elements of information is read aloud to the participant who must then immediately recall as many elements of the story as possible. The participant is told to remember the story for later recall and 20 to 30 min later is asked to recall the story again (a cue is given if necessary). The number of story elements recalled immediately and after the delay interval is recorded. One point is awarded for each element recalled for a maximum total score of 25 for each condition (immediate, delay). The delay interval is filled with a demographic survey and administration of the MoCA.

For those who preferred to be tested in Spanish, the screening battery consisted of Spanish translations of the Mini-Mental State Examination (MMSE [18];) and the Consortium to Establish a Registry for Alzheimer’s Disease (CERAD) Memory Test [19]. Different tests were used for Spanish and English speakers based on the availability of appropriate translations and normative data. The MMSE is a global mental status test that briefly assesses orientation to time and place, word list registration, attention, word list recall, and language. Scores range from 0 to 30, with higher scores indicating better performance. The CERAD Memory Test is a list learning test in which a participant is asked to read aloud and remember 10 common words as they are visually presented one by one, and then to immediately recall as many of the words as possible. This read-and-recall procedure is repeated across three learning trials. After a 5-min delay (filled with a drawing task), the participant is again asked to recall as many words as possible. Finally, a yes-no recognition test is presented for the 10 words randomly interspersed with 10 distractor words.

### Scoring and interpretation

Immediately after cognitive testing, the rater scored the two test measures (e.g., MoCA, story recall), determined *z*-scores using formulas based on age- and education-adjusted normative data, and used the *z*-scores with the algorithm shown in Table 1 to determine the participant’s cognitive classification. An education-adjusted MoCA score less than 24 was used to differentiate abnormal from normal performance based on normative data from general community populations [20, 21]. A Logical Memory delayed recall score at least 1.5 standard deviations below the expected age and education normative score was used to differentiate abnormal from normal performance. Normative data used for this calculation was from the large National Alzheimer’s Coordinating Center Uniform Data Set (NACC UDS) normal sample [22]. Cutoff scores for the MMSE and CERAD Memory Test used for Spanish language testing were 1.5 standard deviations below the expected age- and education-adjusted scores derived from longitudinal data from the UCSD Shiley-Marcos ADRC [23].

**Table 1:** Classification algorithms used for English and Spanish screening procedures. Classification was determined by a combination of performance on a test of delayed recall and a test of global mental status

**English Administration**	Story A delayed recall score		MoCA score
*Normal, Age-Appropriate Memory*	< -1.5 SD		≥ 24
	< -1.5 SD	and	< 24
*Possible Memory Decline*		or	
	≥ -1.5 SD	and	≥ 24
*Likely Memory Decline*	≥ -1.5 SD		< 24
**Spanish Administration**	CERAD delayed recall score		MMSE score
*Normal, Age-Appropriate Memory*	≥ 4		≥ 26
*Possible Memory Decline*	≥ 4	and	< 26
		or	
	< 4	and	≥ 26
*Likely Memory Decline*	< 4		< 26

*MoCA* Montreal Cognitive Assessment, *CERAD* Consortium to Establish a Registry for Alzheimer’s Disease, *MMSE* Mini-Mental State Examination, *SD* standard deviation

Based on application of the algorithm, the participant received immediate written feedback from the rater that stated: “Normal, Age-Appropriate Cognition,” “Possible Decline in Cognition that is greater than expected,” or “Likely Decline in Cognition that is greater than expected.” It was emphasized that this was not a “diagnosis” but simply information that could be used by the participant to prompt interaction with his/her physician.

### Debriefing on results, resources, and research

At the completion of testing, each participant was invited to visit a conference room with refreshments where they could meet with ADRC staff members to ask questions about their testing feedback, receive educational brochures about memory loss and potential causes of memory decline (including AD), and discuss local resources for those with memory concerns (e.g., *Alzheimer’s Association*, *Alzheimer’s San Diego*). Staff members reiterated that the screening result was not a “diagnosis” but simply information that should prompt interaction with their physician to determine “next steps.” The importance of further evaluation to verify memory decline and better understand its causes was emphasized, as was the importance of gaining knowledge about health/lifestyle factors that might reduce dementia risk.

Opportunities to participate in research on memory loss and dementia at the ADRC or affiliated studies were described by staff members. Those interested were invited to review and sign the UCSD IRB approved consent and HIPAA forms that allowed them to be contacted in the future about participating in specific ADRC-affiliated research projects, and permitted their demographic information and screening data to be added to a Research Registry maintained by the ADRC. The Research Registry is used to match participants’ research study preferences and eligibility with specific study requirements. These forms could be completed immediately or taken home so the participant could spend additional time considering whether research participation was an option they would like to pursue. If participants consented to taking part in additional research, they were asked to complete a research interest form which asked about their health history, the likely availability of a study partner (i.e., someone who knows them well enough to provide information about their activities of daily living), and their attitudes towards potential research procedures such as magnetic resonance imaging (MRI), lumbar puncture (LP), and autopsy.

### Statistical analyses

Age and education, MCQ scores, and scores on the cognitive tests were compared across groups (*Normal*, *Possible Decline*, or *Likely Decline*) using one-way analysis of variance (ANOVA) with Tukey’s least-significant difference (LSD) tests for post hoc pair-wise comparisons when ANOVA results were significant. Distributions of the binary categorical variables sex, ethnicity (non-Hispanic, Hispanic), and family history (positive, negative) were compared using 2 × 3 (*Normal*, *Possible Decline*, or *Likely Decline*) chi-square (*χ*^2^) tests followed by 2 × 2 *χ*^2^ tests for post hoc pair-wise comparisons when the 2 × 3 results were significant. All analyses were performed in SPSS version 21.

## Results

### Demographics and cognitive screening

**Fig. 1:**
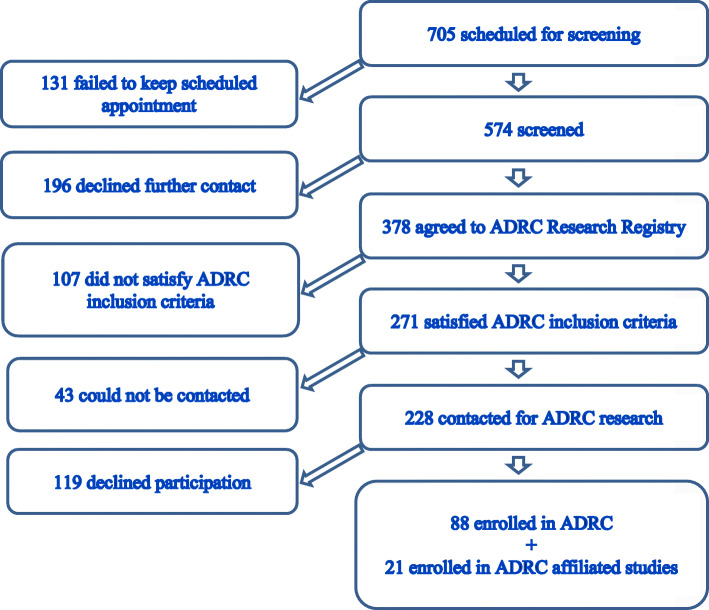
Schematic diagram of participant flow through the screening and recruitment process

**Fig. 2:**
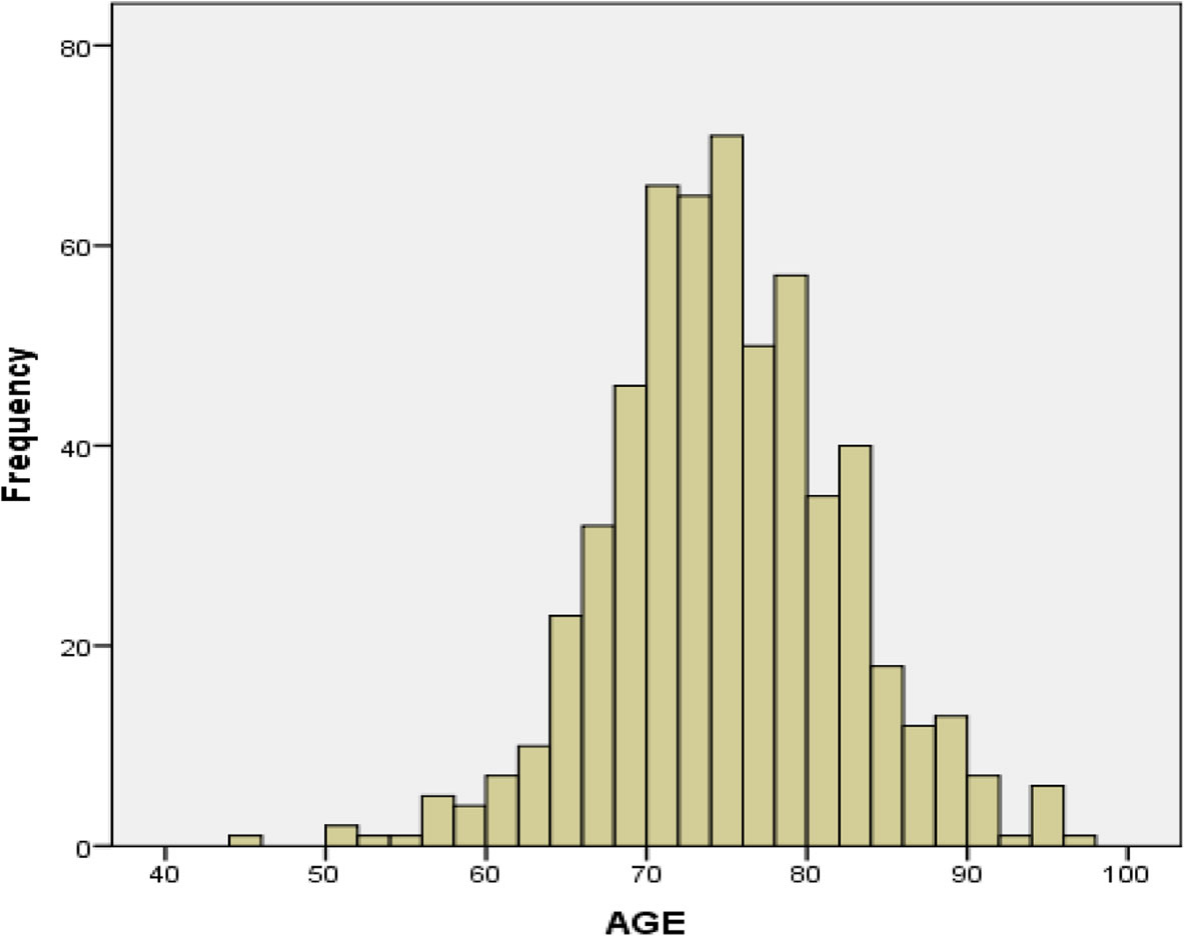
Age distribution of individuals that completed memory screening

A total of 705 individuals were scheduled for screening appointments across the six 2017–2018 Memory Screening Day events (see Fig. 1). One hundred thirty-one individuals (18.6%) failed to keep their scheduled appointment, and 574 participants (81.4%) completed screening. The mean age of those who completed screening was 74.5 years (SD = 7.4; range = 45–96 years), and the mean level of education was 16.1 years (SD = 2.9; range = 0–20 years; education was missing for 6 individuals). There were 252 men (43.9%) and 322 women (56.1%). Although screening was targeted towards those age 65 or older (as indicated in the newspaper article), a small number of younger individuals (*n* = 37) were allowed to participate due to a positive family history of dementia and significant anxiety related to their cognitive function. We decided to leave them in the study for the sake of completeness in describing our true experience in the Memory Screening Day events. The age distribution of those screened is shown in Fig. 2. There were 95 aged 65–69 years (16.6%), 309 aged 70–79 years (53.8%), 118 aged 80–89 years (20.6%), and 15 aged 90 years or older (2.6%). Almost half (46%) of those screened reported a positive family history of dementia (i.e., dementia in a first degree relative), and approximately half (46.4%) answered yes to “concern about memory by yourself or others” on the MCQ (see supplemental Table; this MCQ item was missing for 22 individuals). The average score on the MCQ was 2.2 (SD = 2.3, range = 0–10; total MCQ score was missing for 96 individuals).

Ethnicity/race was reported as non-Latino White for 492 participants (85.7%), Hispanic/Latino for 44 participants (7.7%), Asian-American for 18 participants (3.1%), African-American for 7 participants (1.2%), American Indian/Alaskan Native for 2 participants (0.3%), and Pacific Islander-American for 2 participants (0.3%). Eleven participants (1.9%) declined to state their ethnicity/race. Cognitive testing was administered in Spanish for 19 of the 44 Hispanic/Latino participants (43%) and in English for the remainder of the sample (*n* = 555). The English-speaking participants averaged 23.8 (SD = 4.0; range = 6–30) on the MoCA, 11.4 (SD = 4.4; range = 0–22) on immediate story recall, and 10.0 (SD = 4.8; range = 0–26) on delayed story recall. The 19 participants tested in Spanish averaged 24.4 (SD = 5.7; range = 6–30) on the MMSE, 15.8 (SD = 5.8; range = 0–22) on trials 1–3 total of the CERAD Memory Test, and 5.8 (SD = 2.7; range = 0–26) on delayed recall of the CERAD Memory Test.

Based on our cognitive test score algorithm, 297 participants (52.0%) were classified as “Normal, Age-Appropriate Performance,” 192 (33.6%) as “Possible Memory Decline,” and 82 (14.4%) as “Likely Memory Decline.” Three participants did not complete testing and were not classified. Demographic information for the three classification groups is presented in Table 2. The classification groups differed in age [*F* (2, 568) = 22.77; *p* < .001] with “Likely Decline” older than “Possible Decline” (*p* < .001) and “Normal” (*p* < .001), and “Possible Decline” older than “Normal” (*p* < .001). The groups also differed in education [*F* (2, 565) = 9.13; *p* < .001] with “Likely Decline” (*p* < .008) and “Possible Decline” (*p* < .001) less educated than “Normal,” but not different from each other. The percentage of men differed across the three groups [*χ*^2^ = 23.4; *p* < .001] with the “Likely Decline” group having a higher percentage than the other two groups [vs. “Normal”: *χ*^2^ = 23.4; *p* < .001; vs. “Possible Decline”: *χ*^2^ = 23.4; *p* < .001], and the “Possible Decline” having a higher percentage than the “Normal” group [*χ*^2^ = 4.8; *p* = .03]. There were fewer participants with a positive family history of dementia in the “Likely Decline” group than in the “Possible Decline” [*χ*^2^ = 9.1; *p* = .01] and “Normal” [*χ*^2^ = 10.6; *p* = .005] groups, which did not differ from each other. The percentage of Hispanic/Latino participants differed across groups [*χ*^2^ = 6.1; *p* < .05] with a higher percentage in the “Possible Decline” group than in the “Normal” group [*χ*^2^ = 6.0; *p* = .01] only. The classification groups differed in scores on the MCQ [*F* (2, 475) = 9.86; *p* < .001] with “Likely Decline” higher (i.e., more concerns) than “Possible Decline” (*p* < .01) and “Normal” (*p* < .001), and “Possible Decline” higher than “Normal” (*p* < .03). The percentage of each classification group that endorsed each MCQ item is shown in the supplemental Table.

**Table 2:** Demographic characteristics for the memory screening classification subgroups (upper panel) and cognitive test scores for those tested in English (lower panel). Too few participants were tested in Spanish for separate analyses

	“Normal”	“Possible Decline”	“Likely Decline”
	*n* = 297	*n* = 192	*n* = 82
Age			
Mean	72.8	75.2	78.7
SD	(6.4)	(8.2)	(7.3)
Range	51–95	45–96	60–95
Missing (*n*)	0	0	0
Education			
Mean	16.6	15.5	15.6
SD	(2.4)	(3.4)	(3.1)
Range	8–20	0–20	0–20
Missing (*n*)	1	2	0
Sex (M:F)	108:189	89:103	54:28
Family history +	150 (50.5%)	90 (46.9%)	25 (30.5%)
Hispanic/Latino	16 (5.4%)	22 (11.5%)	6 (7.3%)
MCQ score			
Mean	1.8	2.3	3.2
SD	(2.1)	(2.4)	(2.6)
Range	0–9	0–10	0–10
Missing (*n*)	43	35	15
	“Normal”	“Possible Decline”	“Likely Decline”
	*n* = 292	*n* = 181	*n* = 79
MoCA			
Mean	26.4	21.9	19.0
SD	(2.3)	(3.0)	(3.7)
Range	6–30	11–29	8–23
Missing (*n*)	0	0	0
Story recall immediate			
Mean	13.7	10.3	5.3
SD	(3.1)	(3.7)	(2.8)
Range	6–22	0–21	0–13
Missing (*n*)	0	0	0
Story recall delay			
Mean	12.7	8.6	3.3
SD	(3.5)	(3.7)	(2.4)
Range	5–25	0–20	0–14
Missing (*n*)	0	0	0

*SD* standard deviation, *MCQ* Memory Concerns Questionnaire, *MoCA* Montreal Cognitive Assessment

The classification groups tested in English, as expected, differed in scores achieved on the MoCA [*F* (2, 551) = 282.72; *p* < .001], immediate story recall [*F* (2, 549) = 219.92; *p* < .001], and delayed story recall [*F* (2, 549) = 260.67; *p* < .001] (see Table 2). The three classification groups differed from each other on all three test measures (all *p* values < .01). This was as expected since these tests were used to define the groups, and should not be taken as validation of the tests for identifying cognitive impairment. Groups tested in Spanish were too small for separate analysis (*n* = 5 “Normal,” *n* = 11 “Possible Decline,” *n* = 3 “Likely Decline”).

### Attitudes towards research and specific research procedures

Three hundred seventy-eight of the 574 (66%) screened participants agreed to be included in the ADRC Research Registry and to be contacted about future research participation. Those who agreed and those who declined did not differ in average age [*t* (572) = 1.75, *p* = .08], but those who agreed had more education [*t* (566) = − 3.02, *p* = .003] than those who declined (see Table 3). The groups did not differ in the percentage of females [*χ*^2^ = 1.52, *p* = .22], percentage of those with positive family history [*χ*^2^ = 4.15, *p* = .13], or percentage of Hispanic/Latino individuals [*χ*^2^ = 1.73, *p* = .19]. Those who agreed to additional research scored worse on the MCQ than those who declined [*t* (476) = − 2.71, *p* = .007]. The two groups did not differ on MoCA scores [*t* (550) = − 1.76, *p* = .08], but those who agreed had worse scores on immediate [*t* (550) = − 2.72, *p* = .007] and delayed [*t* (550) = − 2.01, *p* = .045] story recall than those who declined. Different percentages of individuals from the “Normal” (65.0%), “Possible Decline” (72.4%), and “Likely Decline” (52.4%) screening classifications agreed to further research [*χ*^2^ = 10.3, *p* = .006] with “Likely Decline” less likely to agree than “Possible Decline” [*χ*^2^ = 10.2, *p* = .001] or “Normal” [*χ*^2^ = 4.3, *p* = .04].

**Table 3:** Demographic characteristics of screened subjects (*n* = 574) who agreed or declined to be included in the Research Registry and contacted about potential research opportunities (upper panel) and cognitive test scores for those tested in English only (lower panel). Too few participants were tested in Spanish for separate analyses

	Agreed, *n* = 378	Declined, *n* = 196
Age		
Mean	74.1	75.2
SD	(6.9)	(8.4)
Range	54–96	45–95
Missing (*n*)	0	0
Education		
Mean	16.3	15.6
SD	(2.5)	(3.6)
Range	6–24	0–23
Missing (*n*)	5	1
Sex (% female)	57.9%	52.6%
Family history +	186 (49.2%)	79 (40.3%)
Hispanic/Latino	25 (7%)	9 (10%)
MCQ score		
Mean	2.4	1.8
SD	(2.4)	(2.2)
Range	0–10	0–10
Missing (*n*)	64	32
	Agreed, *n* = 371	Declined, *n* = 184
MoCA		
Mean	24.1	23.4
SD	(3.7)	(4.5)
Range	6–30	8–30
Missing (*n*)	0	0
Story recall immediate		
Mean	11.8	10.7
SD	(4.2)	(4.6)
Range	0–22	0–22
Missing (*n*)	0	0
Story recall delay		
Mean	10.3	9.4
SD	(4.6)	(5.1)
Range	0–25	0–25
Missing (*n*)	0	0

*SD* standard deviation, *MCQ* Memory Concerns Questionnaire, *MoCA* Montreal Cognitive Assessment

Those that agreed to be included in the Research Registry (*n* = 378) were asked about their willingness to participate in various AD research procedures and the availability of a study partner. The number of individuals who responded “Yes,” “Maybe,” or “No” to various potential research procedures is presented in Table 4. Overall, 79.1% responded “Yes” or “Maybe” to a research lumbar puncture (LP), 82.0% responded “Yes” or “Maybe” to a research MRI, and 82.0% responded “Yes” or “Maybe” to autopsy. In addition, 77.0% responded “Yes” or “Maybe” when asked if a study partner would be available. More than half of the participants (192/378, 50.8%) agreed to all 3 procedures and reported an available study partner. When we separately examined responses from the 25 Hispanic/Latino participants who completed the questionnaire, 68.0% (17/25) responded “Yes” or “Maybe” to a research LP, 84.0% (21/25) responded “Yes” or “Maybe” to a research MRI, 80.0% (20/25) responded “Yes” or “Maybe” to autopsy, and 72% (18/25) responded “Yes” or “Maybe” when asked if a study partner would be available.

**Table 4:** Number (and percentage) of participants who agreed to be included in the Research Registry (*n* = 378) and responded “Yes,” Maybe,” or “No to being amenable to specific research procedures and to availability of a study partner

	Amenable to lumbar puncture	Amenable to MRI	Amenable to autopsy	Study partner available
Yes*	251 (66.4%)	294 (77.8%)	292 (77.2%)	289 (76.5%)
Maybe	34 (9.0%)	22 (5.8%)	16 (4.2%)	2 (0.5%)
No	48 (12.7%)	25 (6.6%)	18 (4.8%)	23 (6.1%)
No response	45 (11.9%)	37 (9.8%)	52 (13.8%)	64 (16.9%)

*192 (50.8%) were amenable to all 3 procedures and reported available study partner

### Recruitment and enrollment into additional research

The 378 individuals entered into the Research Registry were reviewed to determine if they satisfied inclusion and exclusion criteria for the ADRC longitudinal observational study. One hundred seven (28.3%) did not satisfy these criteria and were not eligible for recruitment into the study. The primary reason for exclusion was related to some aspect of the participant’s health or medical history (e.g., history of stroke, significant psychiatric disorder) that was inconsistent with study criteria (*n* = 63). Other reasons included lack of an available study partner (*n* = 15), young age (*n* = 13), severe cognitive impairment (*n* = 9), infeasible travel distance (*n* = 5), or non-compliance with study procedures (*n* = 2).

Efforts were made to contact the remaining 271 individuals by telephone. Forty-three individuals could not be contacted (11.4%). The remaining 228 were contacted and asked about willingness to enroll in the ADRC longitudinal study after study procedures had been fully described. One hundred nineteen declined to participate and cited unwillingness to comply with one or more required study procedures (*n* = 73), lack of interest (*n* = 35), or a variety of personal reasons (e.g., traveling, family emergency) (*n* = 11). Eighty-eight individuals were successfully enrolled in the ADRC longitudinal study and agreed to all required study procedures (i.e., yearly cognitive testing, baseline LP and MRI, autopsy). Twenty-one were referred directly to affiliated research studies, including clinical trials, and were successfully enrolled. These individuals either made a specific request to join a clinical trial or did not meet all criteria for enrollment in the ADRC longitudinal study. Overall, 19% (109/574) of all screened individuals were successfully recruited into AD research as part of the ADRC longitudinal observational study (88/574, 15.3%) or an affiliated AD-related study or clinical trial (21/574, 3.7%).

### Validation of memory screening classifications

As part of their participation in the ADRC longitudinal study, 86 individuals who had been screened received comprehensive clinical, neurological, and physical evaluations, and a consensus diagnosis (or classification) of dementia (and etiology), mild cognitive impairment (MCI), or cognitively normal (ADRC procedures have been described in detail previously [24]). The ADRC evaluations generally occurred within 6 months after screening. Two of those screened enrolled in the ADRC but did not complete the full set of evaluations. The concordance between screening classification and ADRC “gold standard” diagnosis/classification is shown in Table 5. The overall accuracy of the screening classification was approximately 72%. Accuracy was 88.9% for the “Normal” classification, 36% for the “Possible Decline” classification (if an ADRC diagnosis of either MCI or dementia is considered correct), and 71.4% for the “Likely Decline” classification (if an ADRC diagnosis of either MCI or dementia is considered correct).

**Table 5:** Concordance between memory screening classification and Alzheimer’s Disease Research Center (ADRC) “gold standard” diagnosis/classification

	ADRC Clinical Diagnosis		
	Normal	Mild cognitive impairment (MCI)	Dementia (probable AD)
	*n* = 66	n = 16	*n* = 4
Memory screen classification			
“Normal”	48	5	1
“Possible Decline”	16	8	1
“Likely Decline”	2	3	2

Fourteen of 20 individuals who received an ADRC diagnosis of dementia or MCI had been classified as having “Possible Decline” or “Likely Decline” by screening (i.e., 70% sensitivity). Most of the accurately classified (by screening) truly impaired (by ADRC diagnosis) individuals had been classified as “Possible Decline” by screening (*n* = 9 of 14). Forty-eight of 66 individuals who were considered “Normal” based on the ADRC evaluation were classified as “Normal Cognition” by screening (73% specificity). Most misclassified truly normal individuals (by ADRC diagnosis) had been misclassified as “Possible Decline” by screening (*n* = 16 of 18). The negative predictive value of the memory screening procedure (i.e., no true decline by ADRC evaluation when memory screening classified as normal) was 89%, while the positive predictive value (i.e., true decline by ADRC evaluation when memory screening classified as having decline) was only 44%.

## Discussion

The memory screening and feedback procedures we employed provided a quick and effective way to screen for memory loss and cognitive decline in large numbers of older individuals and offered an opportunity to recruit appropriate participants into AD-related research. Over just 6 single-day events, more than 570 individuals were screened, given information about cognitive decline and AD, and introduced to research. Sixty-six percent (*n* = 378) agreed to participate in research and were added to a Research Registry. Of those added to the Research Registry, 72% (*n* = 271) met inclusion criteria for recruitment into the ADRC longitudinal study. Ultimately, 40% (109/271) of those who met criteria were successfully recruited into the longitudinal study (*n* = 88) or an ADRC-affiliated study or clinical trial (*n* = 21). While the recruitment method is somewhat labor intensive, overall 66% of those screened agreed to participate in clinical research and be contacted about particular studies for which they would meet recruitment criteria. This is far higher than for previously reported recruitment methods that yielded 25–30% agreement to participate in clinical research [25, 26]. Furthermore, 19% of those screened met rigorous inclusion requirements for the UCSD ADRC and its affiliated studies. This level of recruitment is particularly notable since participation in the ADRC longitudinal study required consent to MRI, LP, genotyping, and autopsy. The results suggest that holding a few concentrated events each year may be an effective and relatively efficient recruitment strategy. The success of our memory screening strategy is consistent with findings that community grass-roots outreach events are significantly more productive for recruitment than other methods such as targeting referrals from primary care physicians [26].

The demographic characteristics of the screened participants were similar to those of the current UCSD ADRC longitudinal study cohort and to those reported by the NACC for all US Alzheimer’s Disease Research Centers (https://www.alz.washington.edu/). NACC ADRC values based on more than 40,000 cognitively normal, MCI, and dementia participants (as of September 2019) are 74.7 years of age (on average), 73.4% with at least some college education, 57% female, and 81% white (https://www.alz.washington.edu/WEB/UDSonepage.pdf). Our memory screening sample averaged 74.5 years of age and 16.1 years of education, and was 56.1% female and 92% non-Hispanic white. Thus, our screening procedures draw a sample that is representative of people currently participating in AD research. The high level of education and low percentage of underrepresented minority participants in our sample may be attributable to holding the events at the ADRC site on the UCSD Medical Campus which is located in a largely white, affluent community in San Diego.

The screening procedures classified 52% of the participants as having normal, age-appropriate performance; 34% as having possible memory or cognitive decline; and 14% as having likely memory or cognitive decline. Those classified as having likely memory decline were older than those considered normal, more likely to endorse “concern about memory by yourself or others,” but less likely to have a positive family history of dementia in a first degree relative. These differences could reflect variation in motives for seeking memory and cognitive screening. On the one hand, younger individuals with a positive family history of dementia may be motivated by a generalized anxiety (i.e., the “worried well” [27]) that can be reduced by objective testing. On the other hand, older individuals who have noticed a change in their memory and other cognitive abilities may seek out screening to validate their concerns regardless of family history of dementia.

There were few differences between the screening participants who agreed or refused to participate in additional AD-related research. The two groups were similar in age, education, percentage of females, and percentage of white non-Hispanic individuals. Although family history of AD is a frequent reason for concern that motivates individuals to seek memory screening and more information about AD [28], the two groups were similar in the percentage with a positive family history of dementia in a first degree relative. The groups were also similar in the percentage who reported a “concern about memory by yourself or others” and in the scores they achieved on the objective screening tests. This led to similar percentages in the two groups being classified as having likely, possible, or no memory or cognitive decline. Questions regarding attitudes towards research and specific research procedures could not be explored in those who did not agree to further research, so why some individuals chose to participate in additional research and others did not remains unknown. Because the advertised purpose of the Memory Screening Day event was to provide testing and feedback for individuals, some people may have either been satisfied with that service and not interested in additional research, or may have been curious about research but not willing to commit on the day of the visit. We will investigate these and other possibilities in future studies. In any event, our results suggest that those who agreed and those who refused additional research are equally representative of the population of those screened.

We explored attitudes towards AD research in those who agreed to participate in additional research to determine how amenable they would be to various research procedures and requirements. Most participants indicated that they would likely agree to LP (75%), MRI (84%), and/or autopsy (81%), and more than half (51%) would be amenable to all three. In addition, more than 75% reported that they had or might have a study partner available. Results were essentially the same when limited to Hispanic/Latino participants. Although our subset of Hispanic/Latino participants was small, the results we obtained were similar to the percentages of individuals amenable to LP or autopsy in other underrepresented minority groups [29, 30]. The willingness to participate in these various research procedures was confirmed in the relatively high percentage (40%) of eligible and appropriate screening participants who eventually enrolled in the ADRC longitudinal study or an AD clinical trial where they actually completed an LP and MRI and agreed to autopsy.

The accuracy of the algorithmically derived classification from screening (i.e., “Normal,” “Possible decline,” “Likely decline”) was examined in participants who enrolled in the ADRC longitudinal study and received comprehensive clinical evaluation and diagnosis. While the overall accuracy of the screening procedures was moderate (72% with 70% sensitivity and 73% specificity), they had high negative predictive value (89%) and low positive predictive value (44%). Thus, when screening indicated normal performance (i.e., was “negative”), the participant was also highly likely to be judged as normal by the full ADRC evaluation. In contrast, when screening indicated impaired performance (i.e., was “positive), the participant was not very likely to be judged as impaired by the full evaluation. This was true even though the MoCA cutoff score we employed (i.e., 24) in screening was lower than the recommended cutoff of 26 as “best” for distinguishing MCI and AD from normal age-appropriate memory change [16] and more appropriate for community-dwelling populations without specific memory complaints [20, 21]. The low positive predictive value we achieved indicates that our memory screening procedures were skewed towards “false positive” errors rather than “false negative” errors. There is some debate about the relative risks and benefits of these two types of errors in the memory screening arena [6], but we chose to err on the side of guiding individuals to a more thorough evaluation when they had concerns rather than ignoring a potential problem. We weighed this against the possibility of causing alarm in some cognitively normal individuals and tried to alleviate alarm by emphasizing that the screening results were not a diagnosis but simply information to prompt interaction with their primary healthcare provider.

A number of factors may have contributed to the success of the screening events as a recruitment strategy. First, screening and debriefing were carried out at the ADRC which allowed potential research volunteers to meet and become familiar with the site and ADRC staff. This may have demystified the “ivory tower” image of the academic institution and increased comfort with the idea of returning for similar but potentially more involved research visits. It also provided the opportunity for trust-building that has been cited as an important aspect of successful research recruitment [31]. Second, individuals sought out memory screening when informed of its availability and chose to participate. Thus, they were more likely to have a positive attitude towards screening than individuals who undergo routine cognitive screening as part of a general “wellness visit” with their primary care provider [32]. This may make participation in AD research more palatable if they screen positive for cognitive decline. Third, attending the screening event at the ADRC demonstrated that transportation was not a significant barrier to research participation should they qualify and agree to volunteer. Limited access to transportation is often cited as an important barrier to research participation [33], particularly when individuals are cognitively screened remotely using web-based procedures. Finally, the ability to provide immediate feedback and debriefing after testing may have motivated individuals to consider additional opportunities to participate in research. Access to test results is an important consideration for potential research participants [34], so demonstrating the ability to quickly provide this information and put it in context may have had a positive effect.

Several limitations of our study should be noted. First, we did not collect information about those who scheduled, but failed to attend, an appointment at the Memory Screening Day events. Furthermore, we did not have a telephone informed consent procedure in place, so we did not have permission to use their data (if we had collected any) until after they arrived for their scheduled appointment. We plan to introduce telephone consent to collect and use basic demographic information from the initial telephone contact as our Memory Screening Day program moves forward. Second, there was a low degree of participation by underrepresented minorities in the Memory Screening Day events. We recently expanded our efforts to increase underrepresented minority participation by advertising our Memory Screening Day events in local Spanish language newspapers and offering off-site memory screening in English and Spanish at community centers in Hispanic-majority neighborhoods around San Diego County.

## Conclusions

Conducting a few concentrated community memory screening events each year may help meet the public’s demand for brief assessment of memory concerns and can be a relatively effective and efficient recruitment strategy for AD research.

## Supplementary information

**Additional file 1 : Supplementary material (Table S1)** accompanies this paper.

## Data Availability

Anonymized data and related documents such as study protocols and statistical analysis plans will be shared with any qualified investigator upon request.

## Abbreviations

AD: Alzheimer’s disease
ADRC: Alzheimer’s Disease Research Center
APOE: Apolipoprotein E
CERAD: Consortium to Establish a Registry for Alzheimer’s Disease
HIPAA: Health Insurance Portability and Assurance Act
HRPP: Human Research Protection Program
IRB: Institutional Review Board
LP: Lumbar puncture
MCI: Mild cognitive impairment
MCQ: Memory Concerns Questionnaire
MMSE: Mini-Mental State Exam
MoCA: Montreal Cognitive Assessment
MRI: Magnetic resonance imaging
NACC: National Alzheimer’s Coordinating Center
UCSD: University of California, San Diego
UDS: Uniform Data Set
WMS-R: Wechsler Memory Scale-Revised
