# Supplementary material for: Community memory screening as a strategy for recruiting older adults into Alzheimer’s disease research
Source: Alzheimers Res Ther. 2020 Jun 30;12:78. doi: 10.1186/s13195-020-00643-0 (PMC7325657; doi:10.1186/s13195-020-00643-0)
Supplement: Supplementary file 1 — Additional file 1 : Supplementary material (Table S1) accompanies this paper. [file 13195_2020_643_MOESM1_ESM.docx]

Supplemental Table (S1). The percentage of participants in the memory screening classification subgroups that endorsed each item on the Memory Concerns Questionnaire (MCQ).

|  | | All | | “*Normal*” | | “*Possible Decline*” | | “*Likely Decline*” | |
| --- | --- | --- | --- | --- | --- | --- | --- | --- | --- |
|  | | n=574 | | n=297 | | n=192 | | n=82 | |
| More Forgetful than Others | | 140/536  (26.1%) | | 60/279  (21.5%) | | 52/181  (28.7%) | | 28/76  (36.8%) | |
| More Difficulty Concentrating than Others | | 126/528  (23.9%) | | 51/276  (18.5%) | | 49/178  (27.5%) | | 26/74  (35.1%) | |
| More Difficulty with Routine Tasks than Others | | 71/534  (13.3%) | | 24/276  (8.7%) | | 30/182  (16.5%) | | 17/76  (22.4%) | |
| More Difficulty Recalling Words/Names than Others | | 209/541  (38.6%) | | 93/281  (33.1%) | | 73/182  (40.1%) | | 43/78  (55.1%) | |
| Forget Where You Are Going more than Others | | 58/526  (11.0%) | | 20/274  (7.3%) | | 22/175  (12.6%) | | 16/77  (20.8%) | |
| Misplace Things more often than Others | | 89/523  (16.8%) | | 26/275  (9.5%) | | 44/174  (25.3%) | | 19/74  (25.7%) | |
| Friends/Family Mention Repeating Yourself | | 108/561  (19.3%) | | 47/294  (16.0%) | | 33/189  (17.5%) | | 28/78  (35.9%) | |
| Become Lost While Walking/Driving | | 71/563  (12.6%) | | 32/292  (11.0%) | | 23/189  (12.2%) | | 16/82  (19.5%) | |
| Friends/Family Notice Change in your Mood, Behavior or Personality | | 88/562  (15.7%) | | 35/293  (11.9%) | | 30/188  (16.0%) | | 23/81  (28.4%) | |
| Concern About Memory by Yourself or Others | | 256/552  (46.4%) | | 120/287  (41.8%) | | 79/185  (42.7%) | | 57/80  (71.3%) | |
| Were Changes Gradual or Sudden (% Gradual) | | 494/518  (95.4%) | | 255/264  (96.6%) | | 165/176  (93.8%) | | 74/78  (94.9%) | |
